# Transcriptomic and Functional Evidence Show Similarities between Human Amniotic Epithelial Stem Cells and Keratinocytes

**DOI:** 10.3390/cells11010070

**Published:** 2021-12-27

**Authors:** Li-Ping Liu, Dong-Xu Zheng, Zheng-Fang Xu, Hu-Cheng Zhou, Yun-Cong Wang, Hang Zhou, Jian-Yun Ge, Daisuke Sako, Mi Li, Kazunori Akimoto, Yu-Mei Li, Yun-Wen Zheng

**Affiliations:** 1 Institute of Regenerative Medicine, Affiliated Hospital of Jiangsu University, Jiangsu University, Zhenjiang 212001, China; liuliping@ujs.edu.cn (L.-P.L.); Z.hucheng@ujs.edu.cn (H.-C.Z.); wangyuncong@stmail.ujs.edu.cn (Y.-C.W.); zhouhang@stmail.ujs.edu.cn (H.Z.); micherry159009@outlook.com (M.L.); 2 Department of Dermatology, Affiliated Hospital of Jiangsu University, Zhenjiang 212001, China; 3 Faculty of Medicine, University of Tsukuba, Tsukuba 305-8575, Ibaraki, Japan; D.Zheng@lumc.nl (D.-X.Z.); ge.jianyun.wp@alumni.tsukuba.ac.jp (J.-Y.G.); j3b13049@ed.tus.ac.jp (D.S.); 4 Department of Human Genetics, Leiden University Medical Center, 2333 ZA Leiden, The Netherlands; 5 Department of Obstetrics and Gynaecology, Affiliated Hospital of Jiangsu University, Zhenjiang 212001, China; ladyada828@ujs.edu.cn; 6 Guangdong Provincial Key Laboratory of Large Animal Models for Biomedicine, School of Biotechnology and Health Sciences, Wuyi University, Jiangmen 529020, China; 7 Department of Medicinal and Life Sciences, Faculty of Pharmaceutical Sciences, Tokyo University of Science, Noda 278-8510, Japan; akimoto@rs.tus.ac.jp; 8 School of Medicine, Yokohama City University, Yokohama 236-0004, Kanagawa, Japan

**Keywords:** amniotic epithelial stem cells, keratinocytes, transcriptomics, stemness, mesenchymal, TP63, reprogramming, cell fate, skin substitutes, skin regeneration

## Abstract

Amniotic epithelial stem cells (AESCs) are considered as potential alternatives to keratinocytes (KCs) in tissue-engineered skin substitutes used for treating skin damage. However, their clinical application is limited since similarities and distinctions between AESCs and KCs remain unclear. Herein, a transcriptomics analysis and functional evaluation were used to understand the commonalities and differences between AESCs and KCs. RNA-sequencing revealed that AESCs are involved in multiple epidermis-associated biological processes shared by KCs and show more similarity to early stage immature KCs than to adult KCs. However, AESCs were observed to be heterogeneous, and some possessed hybrid mesenchymal and epithelial features distinct from KCs. A functional evaluation revealed that AESCs can phagocytose melanosomes transported by melanocytes in both 2D and 3D co-culture systems similar to KCs, which may help reconstitute pigmented skin. The overexpression of TP63 and activation of NOTCH signaling could promote AESC stemness and improve their differentiation features, respectively, bridging the gap between AESCs and KCs. These changes induced the convergence of AESC cell fate with KCs. In future, modified reprogramming strategies, such as the use of small molecules, may facilitate the further modulation human AESCs for use in skin regeneration.

## 1. Introduction

Skin damage is a major cause of global disease burden, affecting millions worldwide [1]. Self-repair ability is completely disabled in deep skin injuries affecting large areas such as in burns. Furthermore, extensive wound exposure can cause infection, ultra-high metabolism, and internal environment disorders, resulting in sepsis, multiple organ failure, and even death. Skin-barrier restoration through skin transplantation helps to close skin defects, reconstruct its function, and greatly improve patient survival. However, autologous skin sources are extremely limited and cannot satisfy the high demand. Tissue-engineered skin substitutes can be used to treat acute and chronic skin wounds and can enhance wound healing, reduce inflammatory response, and provide safe coverage [2]. Keratinocytes (KCs), which are primary cells of the epidermis in human skin, are commonly used in tissue engineered skin substitutes and function to synthesize keratin, exerting a protective role [3]. However, autologous KCs are quite limited in quantity and require a long time to prepare and be used as skin substitutes in patients with skin damage. Although the usage of allogeneic KCs can address these issues, other factors such as graft rejection and possible disease transmission still hinder the development and application of skin substitutes.

Amniotic epithelial stem cells (AESCs), located in the inner layer of the amniotic membrane, possess embryonic stem cell-like proliferation and differentiation capabilities [4,5]. They have been demonstrated as useful in regeneration medicine, owing to their accessibility, high cell yield [6], multilineage differentiation potential [7], immune tolerance [8,9], and no tumorigenic features [10]. In animal models of various diseases in the brain, liver, lung, heart, or the ovary, human AESCs have been shown to exert their regenerative potentials through different molecular mechanisms [11]. Furthermore, patients with ovarian insufficiency or bronchial fistula are being recruited in registered clinical trials to assess the safety and effectiveness of allogeneic AESCs transplantation [11], indicating the therapeutic potential of AESCs.

In addition to the abovementioned qualities, AESCs also exert potential activity in skin repair and regeneration due to their epithelial properties. In 1985, Regauer et al. showed that AESCs expressed Keratin (KRT) 1, 4, 5, 6, 8, 10, 11, 14, 17, 18, and 19 in situ and this cytokeratin expression pattern was very similar to that expressed in KCs [12]. Following this, several studies attempted to employ AESCs to replace KCs to establish 3D skin substitutes in vitro [13,14,15,16]. It has been found that AESCs express specific markers of KCs such as KRT14 [13,14,15], and they can also form continuous layers of stratified epithelium similar to those in normal human skin [13,15,16]. In addition, desmosomes, hemidesmosomes, and basement membrane zones, which are all critical structures of the human epidermis, have been detected ultrastructurally in skin substitutes constructed using AESCs [13]. Upon transplantation into immunodeficient mice, tissue-engineered skin constructed with AESCs has been reported to repair full-thickness skin defects [16]. All these results demonstrate the potential of AESCs as an alternative for KCs in skin substitute constitution.

The distinct characteristics of AESCs and KCs are also obvious. The biological characteristics of AESCs have been previously evaluated using conventional approaches such as PCR [5], two-dimensional gel electrophoresis [12], immunostaining [17], and flow cytometry [4]. However, no direct comparison and comprehensive understanding of AESCs and KCs at the whole transcriptome level have been performed. Therefore, the similarities and distinctions between AESCs and KCs remain unclear, which has undoubtedly limited the clinical application of AESCs as the alternative cell source for KCs. Additional details are required to guarantee the efficacy and safety of AESCs during transplantation for cellular therapy in the future. Thus, this study was conducted to understand AESCs more extensively, by employing a transcriptome analysis and functional experiments, which could provide references for the application of AESCs in skin regeneration. 

## 2. Materials and Methods

### 2.1. Tissue Collection and Cell Culture 

Amnion tissues were collected after full-term cesarean section and pre-treated with 0.2 mg/mL egtazic acid. Later, the amniotic membranes were transferred to a 0.05% Trypsin-EDTA (Gibco, 25300062, Carlsbad, USA) solution and incubated for 40 min at 37 °C; this process was repeated twice. Two lots of digests were neutralized and pooled. Cells were centrifuged and resuspended in a medium prepared for AESCs cultivation, which contained 10 ng/mL epidermal growth factor (Sigma, E9644, St. Louis, MO, USA), 5 µg/mL insulin (Wako, 096-03443, Osaka, Japan), 0.5 µg/mL epinephrine (Sigma, E4250, St. Louis, MO, USA), 36 ng/mL hydrocortisone (Wako, 086-10191, Osaka, Japan), 5 µg/mL transferrin (Sigma, T8158, St. Louis, MO, USA), 4 pg/mL triiodo-L-thyronine (Sigma, T2877, St. Louis, MO, USA), and 5% fetal bovine serum (Gibco, 10270-106, Carlsbad, CA, USA).

Adult foreskins were collected after circumcision and were cut into strips and transferred to 0.35 mg/mL Dispase II (Sigma, D4693, St. Louis, MO, USA) solution. The tissues were incubated overnight at 4 °C. To obtain keratinocytes, the epidermis was separated, cut into small pieces, and incubated in 0.25% Trypsin-EDTA (Gibco, 25200072, Ottawa, ON, Canada) for 12 min at 37 °C. Digestion was neutralized and the suspension was filtered through a 100 μm cell strainer. The collected cells were centrifuged, and the cell pellet was resuspended in Epilife medium (Gibco, MEPI500CA, Carlsbad, CA, USA) supplemented with HKGS (Gibco, S0015, Carlsbad, CA, USA). The cells were counted and seeded into culture dishes treated with iMatrix 511 (Matrixome, 892011, Osaka, Japan) at a density of 9 × 10^4^/cm^2^. The protocol used for fibroblast isolation was the same as described in our previous study [18]. Briefly, the separated dermis was cut into smaller pieces and transferred into 10 cm dishes. A stainless steel mesh (Cellamigo^®^, Osaka, Japan) was used to protect the tissue from floating. A culture medium consisting of Dulbecco’s Modified Eagle Medium/high glucose, 10% fetal bovine serum, and 1% penicillin/streptomycin was subsequently added. Fibroblasts were harvested when most colonies reached 90% confluency and then passaged.

Fetal back skin was collected from aborted fetus after termination of pregnancy (at 24 weeks gestation) and embedded in Tissue-Tek^®^ O.C.T. Compound (Sakura, 25608-930, Torrance, CA, USA).

### 2.2. Three Dimensional Skin Equivalent Preparation

Rat tail collagen I (Corning, 354236, Bedford, USA) was diluted (0.8 mg/mL) and applied to the Alvetex Scaffold (12-well format, Reprocell, Durham, UK), which was then kept at room temperature (~20–26 °C) for 1 h. Fibroblasts were seeded on the collagen-coated Scaffold membrane (1 × 10^6^ cells/well) and settled at 37 °C for 1 h. The fibroblast medium was added to a total of 10.5 mL per well from below. The medium was changed every 2–3 days to allow the formation of a dermal equivalent. After one week, melanocytes were seeded with AESCs or KCs (total number 1.5 × 10^6^, ratio 1:5). The medium volume was 500 µL in the insert and about 6.4 mL on the outer side, and the medium in the scaffold was changed every day. After three days, the medium was removed and 4 mL of the differentiation medium was added to each well, such that the bottom of the insert remained in contact with the medium while the upper surface remained exposed to air. The differentiation medium contained 10^−10^ M cholera toxin (Sigma, C8052, St. Louis, MO, USA), 10 ng/mL epidermal growth factor, 0.4 μg/mL hydrocortisone, 5 μg/mL insulin, 5 μg/mL transferrin, 2 × 10^−11^ M triiodo-L-thyronine, and 10% fetal bovine serum. The medium was changed every two days to allow the formation of a full thickness skin equivalent. After two weeks, the 3D skin was embedded in Tissue-Tek^®^ O.C.T. Compound.

### 2.3. Lentiviral Transduction

AESCs were seeded at a density of 1.2 × 10^4^/cm^2^ in 12-well plates, 24 h prior to viral infection. On the second day, the cells were transduced with lentivirus carrying TP63-ZsGreen (HANBIO, Shanghai, China, MOI:80) with polybrene (HANBIO, HB-PB-500, Shanghai, China, 4 μg/mL). The medium was replaced with fresh culture medium after 24 h.

### 2.4. Activation of Notch Signaling Using Jagged-1

Tissue culture plates were pre-coated with 20 µg/mL anti-Human IgG Fc antibody (Sigma, I8885, St. Louis, MO, USA) in phosphate-buffered saline (PBS) at 4 °C overnight and washed twice with PBS. The wells were then treated overnight with 10 µg/mL of recombinant human Jagged 1-Fc (RD, 1277-JG, Minneapolis, MN, USA) at 4 °C, and then washed twice with PBS. The wells coated with anti-Human IgG Fc antibody alone were used as controls. AESCs were then seeded onto the coated surfaces and cultured for 72 h.

### 2.5. Animal Experiments

Male nude (BALB/c Nude) mice (5–7 weeks old, Model Animal Research Center of Nanjing University, Nanjing, China), and neonatal BALB/c mice (1–2 days after birth, Cavens, Changzhou, China) were used as models. The protocols used for skin construction in vivo are outlined in our previous study [18]. Briefly, truncal skins from neonatal BALB/c mice were isolated and incubated in 0.25% trypsin (Gibco, 15050065, Scotland, UK) at 4 °C overnight. On the next day, the dermis was separated and incubated in 0.35% collagenase type I (Gibco, 17100017, Carlsbad, CA, USA) at 37 °C for 40 min to isolate fibroblasts. Mouse fibroblasts mixed with human AESCs or KCs were injected through the chambers in the back skin of nude mice. For each injection, 5 × 10^6^ mouse fibroblasts and 3 × 10^6^ human AESCs or KCs were used. The chambers were removed on the seventh day after grafting. At the third week, the reconstituted tissues were harvested and embedded in Tissue-Tek^®^ O.C.T. Compound.

### 2.6. RNA Sequencing

Total RNA was extracted using TRIzol reagent (Thermo Fisher Scientific, 15596018, Waltham, MA, USA) from AESCs, KCs, and fibroblasts, and RNA sequencing was performed by Eurofins Genomics K.K. (Tokyo, Japan), and the Illumina HiSeq 2500 (Illumina, Santiago, MN, USA) was used.

### 2.7. Bioinformatic Analysis

All paired-end reads of each sample were trimmed using fastp 0.18.0. Then, HISAT2 2.1.0 was used to map filtered reads against the human reference sequence (hg 38). The mapped reads were counted, and transcript abundance was measured in FPKM (fragments per kilobase of transcript per million fragments mapped) units using StringTie (v1.3.4d).

Differentially expressed (fold change of FPKM value >2 or <0.5) gene profiles between AESCs and fibroblasts, and between KCs and fibroblasts were normalized (Z-score normalized FPKM values). Heatmaps were generated using the pheatmap package in R. Each row was scaled to compare the expression of each gene across all samples. The color bar in heatmaps indicated the Z-score. Intersection analysis for AESCs and KCs was performed using the UpSetR package in R. Critical transcription factors (TFs) for KCs were determined using the Cistrome Data Browser. Overlap R was used for the TFs and pathway intersection analysis.

### 2.8. Real-Time PCR

Total RNA was isolated using TRIzol and reverse transcribed to cDNA using a RevertAid First Strand cDNA Synthesis Kit (Thermo Fisher Scientific, K1622, Lithuania) according to the manufacturer’s instructions. Amplification was performed on a real-time PCR system (Applied Biosystems QuantStudio 3, Singapore). The entire procedure was performed according to the manual of the SYBR Premix Ex Taq kit (Takara, RR420A, China). Gene expression levels were normalized to those of GAPDH and quantified based on the Delta Ct method.

### 2.9. Immunocytochemistry

Cells or frozen tissue samples were fixed with 4% paraformaldehyde for 15 min at 4 °C, and then washed and permeabilized thrice using PBST (0.5% Tween20 in PBS) (5 min each time). Normal goat serum (Jackson ImmunoResearch Labs, 005-000-121, West Grove, PA, USA) was used as the blocking reagent for 1 h at room temperature (~20–26 °C) to prevent nonspecific antibody binding. After incubation with primary antibodies overnight at 4 °C, the samples were incubated with the secondary antibody for 1 h. The dilution ratios for the primary antibodies are as follows: KRT14 (Abcam, ab7800, Cambridge, UK, 1:200), KRT10 (Abcam, ab9025, Cambridge, UK, 1:500,), KRT7 (Dako, M7018, Glostrup, Denmark, 1:50), KRT19 (Biolegend, 628506, San Diego, CA, USA, 1:100), CDC20 (Santa Cruz, SC-13162, Dallas, TX, USA, 1:100), PCLAF (Santa Cruz, SC-390515, Dallas, TX, USA, 1:100), PTTG1 (Sigma, HPA008890, St. Louis, MO, USA, 1:100), VIM (DAKO, M0725, Glostrup, Denmark, 1:100), Anti-Nuclei antibody specific for human (Millipore, MAB1281, Temecula, CA, USA, 1:200), gp100 (DAKO, M0634, Carpinteria, CA, USA, 1:100), and TYRP1 (Millipore, MABC592, Darmstadt, Germany, 1:200). The secondary antibodies included Cy3 Goat Anti-Mouse IgG3 (Jackson ImmunoResearch Labs, 115-165-209, USA), Alexa Fluor 488 Goat anti-Mouse IgG1 (Thermo Fisher Scientific, A21121, Waltham, MA, USA), Alexa Fluor 488 Goat anti-Mouse IgG2a (Thermo Fisher Scientific, A21131, Waltham, MA, USA), Cy3 Goat Anti-Rabbit IgG (Jackson ImmunoResearch Labs, 111-165-144, West Grove, PA, USA), and Alexa Fluor 647 Goat anti-Mouse IgG1 (Thermo Fisher Scientific, A-21240, Waltham, MA, USA). The dilution ratio for all secondary antibodies was 1:500. The mean fluorescent intensity of each image was calculated using ImageJ.

### 2.10. Statistical Analysis

Data of real-time PCR are presented as mean ± SD. The Student’s *t* test was used to calculate the *p* values where appropriate. *p* < 0.05 was considered to be significant.

## 3. Results

### 3.1. Similarities between AESCs and KCs in Epidermis-Associated Biological Processes by Transcriptomics

**Figure 1:**
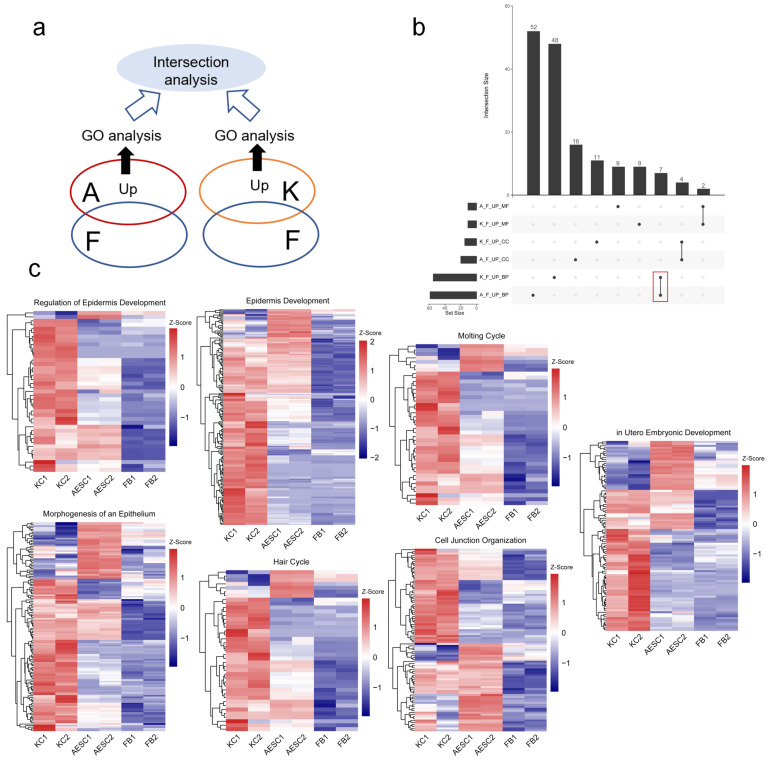
Transcriptomics reveals similarities between amniotic epithelial stem cells (AESCs) and keratinocytes (KCs) in epidermis-associated biological processes. (**a**) Strategy for similarity analysis of transcriptomics. Upregulated and differentially expressed genes in AESCs (A) and KCs (K) were screened by comparing them with fibroblasts (F) for further gene ontology (GO) analysis and intersection analysis; (**b**) Intersection analysis of involved GO terms of highly upregulated genes in AESCs (A) and KCs (K) compared to those in fibroblasts (F) revealed 7, 4, and 2 terms associated with biological process (BP), cellular component (CC), and molecular function (MF), respectively. (**c**) Heatmaps of genes involved in seven intersected BP terms between AESCs and KCs. F & FB, fibroblast; A & AESC, amnion epithelial stem cell; K & KC, keratinocyte.

To understand the similarities between human AESCs and KCs in detail, we performed RNA sequencing of AESCs, KCs, and fibroblasts and analyzed their transcriptome features (Figure 1a). Fibroblasts, another group of commonly found cells in the skin, were used as the control. Upregulated and differentially expressed genes in AESCs and KCs were screened by comparing them with fibroblasts for further gene ontology (GO) analysis. The upregulated genes in AESCs were significantly enriched in 52, 16, and 9 GO terms associated with biological process (BP), cellular component (CC), and molecular function (MF), respectively. The corresponding number of enriched GO terms for the upregulated genes in KCs was 48, 11, and 9, respectively (Figure 1b). For the similarity evaluation, intersection analyses between these corresponding groups were performed, which revealed 7, 4, and 2 terms associated with BP, CC, and MF, respectively, which were shared by both AESCs and KCs (Figure 1b, Table 1). Interestingly, six of the seven intersected terms in BP were related to epidermis development, morphogenesis of an epithelium, regulation of epidermis development, cell junction organization, molting cycle, and hair cycle, respectively. To further verify the similarity between AESCs and KCs in these processes, the expression of genes involved in each of the seven intersected BP terms was visualized. Heatmaps indicated that most genes in these epidermis-associated terms were highly expressed in both KCs and AESCs when compared to fibroblasts (Figure 1c and Figure S1, Table S1).

Overall, the transcriptome analysis revealed the similarity between AESCs and KCs in molecular features, especially in epidermis-associated biological processes.

### 3.2. Similarity of AESCs with Early Stage Immature KCs

**Figure 2:**
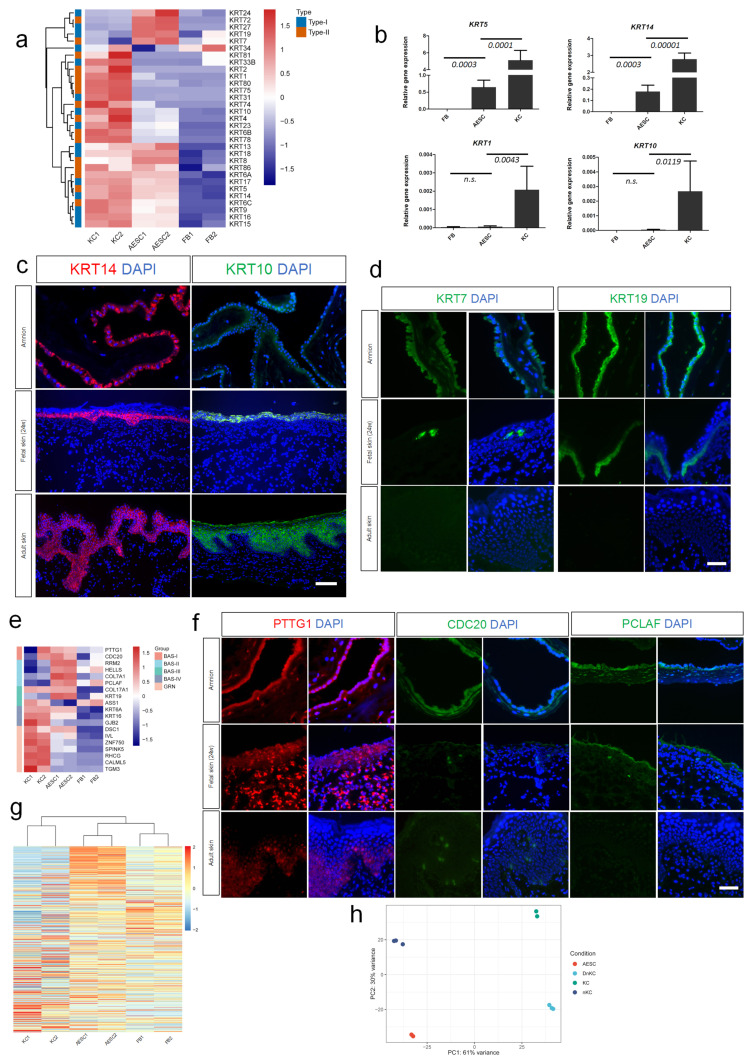
Similarity of amniotic epithelial stem cells (AESCs) to early stage immature keratinocytes (KCs). (**a**) Heatmap of keratin family genes revealed specific and distinct patterns in AESCs and KCs. (**b**) Expression of undifferentiated (*KRT5* & *KRT14*) and differentiation (*KRT1* & *KRT10*) genes in AESCs detected by qRT-PCR. (**c**) KRT14 and KRT10 immunostaining in the amnion, fetal skin (at 24 weeks gestation) and adult foreskin. (**d**) KRT7 and KRT19 immunostaining in the amnion, fetal skin (at 24 weeks gestation) and adult foreskin. (**e**) Heatmap reveals that critical markers of early stage KCs were highly expressed in AESCs. (**f**) PTTG1, CDC20, and PCLAF immunostaining in the amnion, fetal skin (at 24 weeks gestation) and adult foreskin. (**g**) Heatmap of pseudotime-dependent genes involved in keratinocyte differentiation (Wang et al. [22]) shows that genes enriched in early stage KCs were highly expressed in AESCs (top to bottom corresponds to early stage to late stage). (**h**) Principal component analysis indicated that AESCs were closer to undifferentiated neonatal KCs (nKC). FB, fibroblast; AESC, amnion epithelial stem cell; KC, (adult) keratinocyte; nKC, neonatal KC; DnKC, differentiated neonatal KC (induced by adding 1.2 mM CaCl_2_) [23]. Bars represent relative values (mean ± SD) calculated from at least three independent experiments. Student’s *t* test was used to calculate *p* values. Scale bar = 100 μm (**c**); 50 μm (**d**,**f**).

Keratins, the cytoskeletal filament-forming proteins in the epidermis and other epithelial tissues, demonstrated highly specific expression patterns depending on the epithelial type and the stage of cellular differentiation [19]. Using transcriptome analysis, specific and distinct patterns of keratin expression were also found in AESCs and KCs (Figure 2a). For instance, the differentiation and keratinization markers of KCs, KRT1 and KRT10 were lowly expressed in AESCs (Figure 2a–c and Figure S2a), indicating that AESCs did not show differentiation features. However, AESCs and KCs shared intersections such as KRT5 and KRT14. Both qRT-PCR and immunostaining further confirmed the high expression of these keratins in both AESCs and KCs (Figure 2b,c and Figure S2a).

According to Figure 2a, when compared to KCs, AESCs showed high expressions of a distinct group of keratins, including *KRT24*, *KRT72*, *KRT27*, *KRT19*, and *KRT7*. Although *KRT7* and *KRT19* were hardly detected in KCs (isolated from adult skin), their expression has been identified in fetal epidermis in previous studies [20,21]. To verify this, KRT7 and KRT19 were assessed by immunostaining, which confirmed their expression in both fetal skin and amnion, but not in adult skin (Figure 2d and Figure S2b). Thus, the keratin expression pattern in AESCs seemed closer to that in fetal KCs compared to that in adult KCs.

KCs showed distinct features during the skin development and differentiation process [21]. Using single cell-RNA sequencing, Wang et al. identified four distinct populations (Bas-I–Bas-IV) of basal keratinocyte stem cells in human neonatal skin and differentiation pseudo-time trajectory revealed the populations of Bas-I and II to be early stage stem cells [22]. To assess whether AESCs showed more similarities to early stage KCs, marker genes identified for each stem cell population in the above mentioned study were used for comparative analysis. Surprisingly, the critical markers of early stage KCs, such as PTTG1, CDC20 (markers for Bas-I), and PCLAF (marker for Bas-II), were highly expressed in AESCs (Figure 2e,f and Figure S2c). AESCs also expressed parts of the defined markers of Bas-III (such as *COL17A1* and *KRT19*) and IV (such as *KRT6A*) clusters; however, they rarely expressed markers of the granular layer of KCs, which represented more differentiated features. In addition, the expression of 700 pseudotime-dependent genes that were identified to describe the keratinocyte differentiation trajectory [22] was visualized. Interestingly, the genes enriched in early stage KCs were highly expressed in AESCs, whereas the later-stage genes were relatively enriched in adult KCs (Figure 2g), indicating the similarity of AESCs to early stage KCs. To further verify this, we compared our data with a dataset of neonatal KCs reported previously [23]. A principal component analysis (PCA) revealed that compared to adult KCs and differentiated neonatal KCs, AESCs were more similar to undifferentiated neonatal KCs according to PC1 (61% variance) (Figure 2h).

Together, the above data indicated that AESCs show more similarities to early stage immature KCs when compared to adult ones.

### 3.3. Differences of AESCs from KCs in Mesenchymal Characteristics

**Figure 3:**
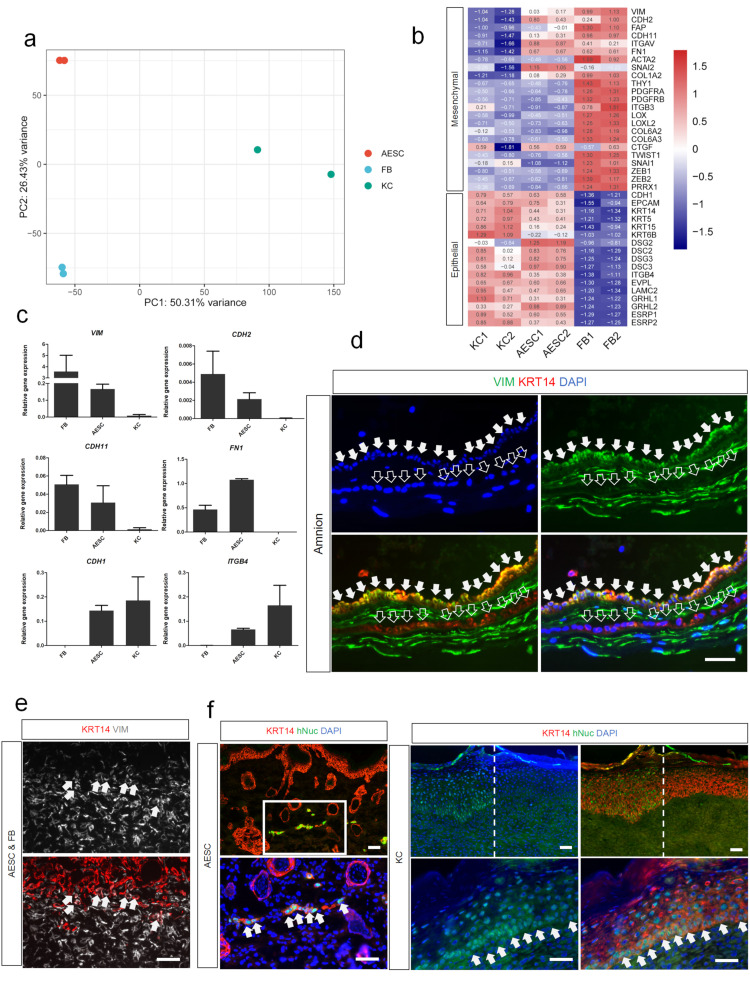
Difference between amniotic epithelial stem cells (AESCs) and keratinocytes (KCs) in mesenchymal characteristics. (**a**) Principal component analysis of AESCs, KCs, and FBs. (**b**) Heatmap showing that both mesenchymal genes and epithelial genes were highly expressed in AESCs. (**c**) qRT-PCR analysis of mesenchymal (*VIM*, *CDH2*, *CDH11*, *FN1*) and epithelial (*CDH1*, *ITGB4*) genes. (**d**) VIM (green) and KRT14 (red) immunostaining in the amnion. Arrows: VIM^+^KRT14^+^ cells; Empty arrows: VIM^−^KRT14^+^ cells. (**e**) VIM (grey) and KRT14 (red) immunostaining in reconstructed 3D skin using AESCs and FBs. Arrows: VIM^+^KRT14^+^ cells. (**f**) Immunostaining for human nuclei (hNuc, green) and KRT14 (red) in reconstituted skin using AESCs and KCs, respectively. Arrows: hNuc^+^KRT14^+^ cells. FB, fibroblast; AESC, amnion epithelial stem cell; KC, keratinocyte. Bars represent relative expression normalized to GAPDH (mean ± SD) calculated from at least three independent experiments. Scale bar = 50 μm (**d**,**e**,**f**).

To further understand the features of AESCs, the transcriptomes of AESCs, KCs, and fibroblasts were compared using a PCA plot. Surprisingly, a similarity between AESCs and FBs was observed according to PC1 (50.31% variance) (Figure 3a). To investigate whether AESCs also exhibited mesenchymal properties in addition to epithelial features, mesenchymal genes identified in skin squamous cell carcinomas [24] were investigated. The heatmap showed that some of the mesenchymal genes such as *VIM*, *CDH2*, *CDH11* and *FN1*, were highly expressed in AESCs when compared to KCs (Figure 3b), and these results were further confirmed by qRT-PCR (Figure 3c). Furthermore, immunostaining revealed that some AESCs in the amnion co-expressed epithelial (KRT14) and mesenchymal (VIM) markers (Figure 3d). This co-expression was also found in the 3D skin equivalent constructed using AESCs (Figure 3e). Thus, these results identified the hybrid epithelial and mesenchymal features of AESCs, and also revealed their heterogeneity.

As mesenchymal cells tend to migrate, AESCs were transplanted into BALB/c nude mice using a skin reconstitution assay to assess this feature, and KCs were used for comparison (Figure S3a). Three weeks later, complete skin repair could be observed in both groups with no significant difference by macroscopic observation (Figure S3b). Reconstituted skin samples were collected and antibodies specific to the human nucleus were used to localize human-origin cells. In the KC group, large amounts of human-origin cells that also expressed KRT14 were found in the stratified epidermis, indicating a high integration of KCs in the epidermis. In contrast, human AESCs expressing KRT14 were only found in the dermis and formed a sheet-like structure (Figure 3f and Figure S2d). These revealed that AESCs and KCs have distinct patterns after transplantation and indicated that AESCs have a higher migration tendency compared to KCs.

Overall, these data indicated the differences between AESCs and KCs in mesenchymal characteristics and further revealed the heterogeneity of AESCs.

### 3.4. AESCs Interact with Melanocytes and Phagocytose Melanosomes

**Figure 4:**
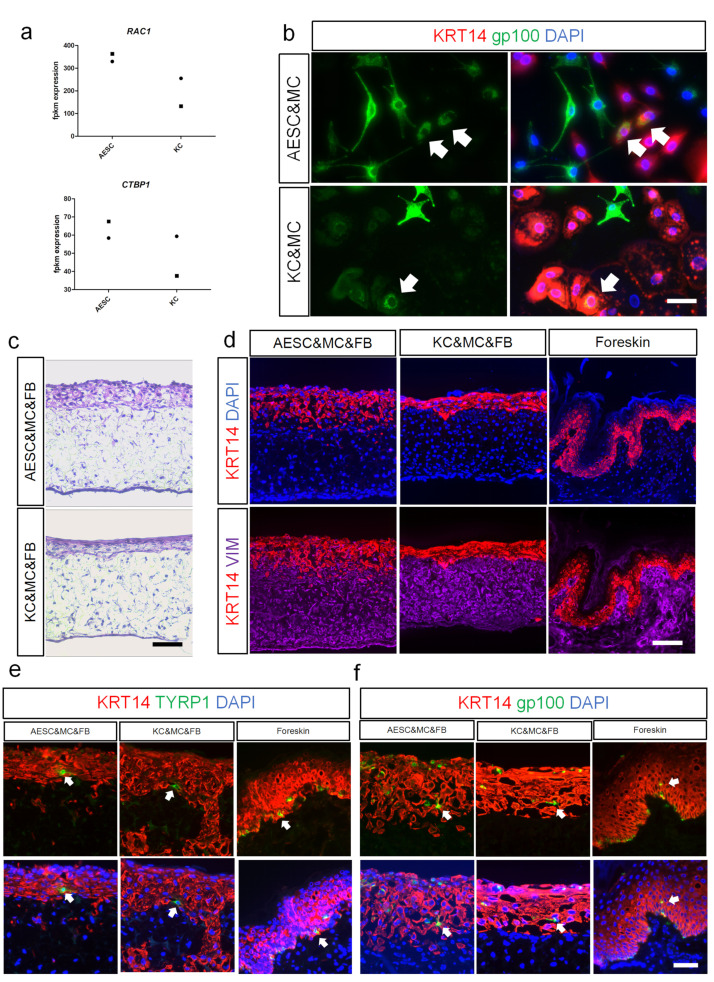
Amniotic epithelial stem cells (AESCs) interact with melanocytes and phagocytose melanosomes. (**a**) *RAC1* and *CTBP1* expression in AESCs and KCs, observed by RNA sequencing. (**b**) KRT14 (red) and gp100 (green) immunostaining in co-culture systems. Arrows: KRT14^+^gp100^+^ cells. (**c**) Reconstructed 3D skin using human fibroblasts, MCs and AESCs or KCs, respectively. (**d**) KRT14 (red) and VIM (purple) immunostaining in reconstructed 3D skin and normal human skin. (**e**) KRT14 (red) and TYRP1 (green) immunostaining in reconstructed 3D skin and normal human skin; Arrows: KRT14^+^TYRP1^+^ cells. (**f**) KRT14 (red) and gp100 (green) immunostaining in reconstructed 3D skin and normal human skin; Arrows: KRT14^+^gp100^+^ cells. FB, fibroblast; AESC, amnion epithelial stem cell; KC, keratinocyte; MC, melanocyte; Scale bar = 100 μm (**c**,**d**); 50 μm (**b**,**e**,**f**).

Skin pigmentation is crucial for protecting the body against ultraviolet irradiation and is a consequence of melanin transport from melanocytes to the surrounding KCs. Thus, melanin transfer is an important interaction in the melanocyte-keratinocyte communication. Recently, Rac1 and CtBP1/BARS were found to be involved in the melanin uptake mechanisms of KCs [25]; interestingly, our sequencing results also revealed their expression in both AESCs and KCs (Figure 4a). To investigate whether melanin transfer also occurs between melanocytes and AESCs, a co-culture system was established. Immunostaining showed that gp100, a specific melanosome marker [26,27], was detected in the peri-nuclear regions of AESCs and co-localized with KRT14 (Figure 4b and Figure S2e). This indicated that AESCs phagocytosed melanosomes that are secreted by melanocytes, similar to the interaction between melanocytes and KCs. To verify whether this phenomenon also existed in 3D skin equivalents, AESCs were cocultured with melanocytes using a scaffold. Meanwhile, fibroblasts were used as the dermal component to facilitate the development of a permissive microenvironment conducive to long-term culture of skin equivalent [28]. After 14 days of culture at the air-liquid interface, a skin equivalent was formed that showed the clear structure of epidermis and dermis (Figure 4c,d and Figure S2f). Both KRT14 and VIM were highly expressed in the reconstructed epidermis and dermis, respectively, similar to the expressions in normal foreskin (Figure 4d and Figure S2f). TYRP1^+^ melanocytes were also detected in the epidermis (Figure 4e and Figure S2g). Co-localization of KRT14 and gp100 was found in both the AESC and KC groups, indicating a successful melanin transfer, which is similar to the phenomenon observed in the 2D co-culture system (Figure 4f and Figure S2g).

These results verified the ability of AESCs to reconstitute pigmented 3D skin equivalents when cocultured with human melanocytes and fibroblasts. AESCs could also interact with melanocytes and phagocytose the transferred melanosomes in both 2D and 3D co-culture systems.

### 3.5. TP63 Enhancement and NOTCH Activation to Induce Cell Fate Convergence of AESCs to KCs

**Figure 5:**
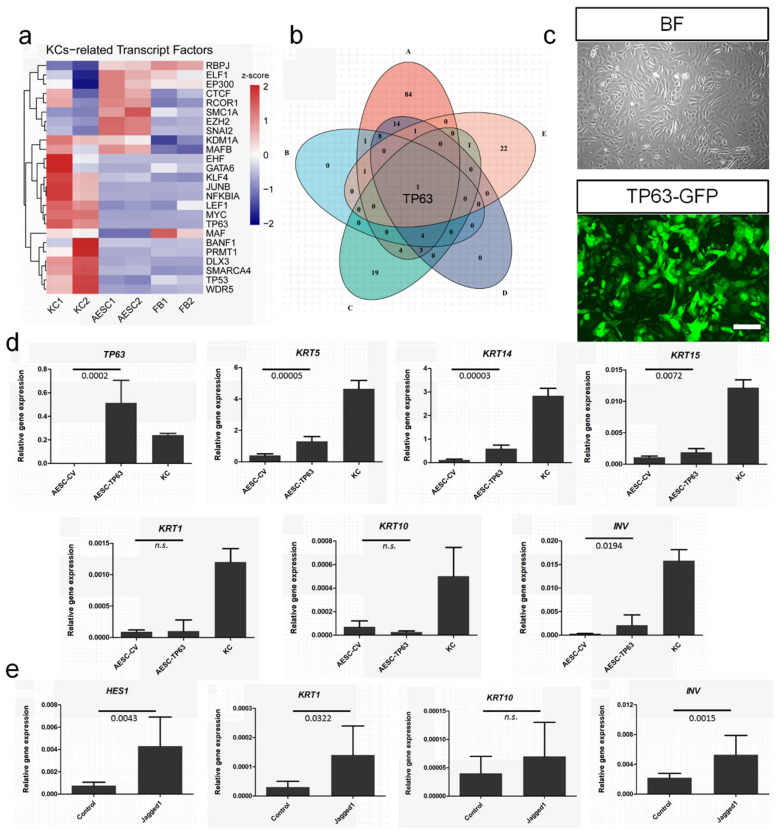
Illustrating TP63 enhancement and NOTCH activation to induce cell fate convergence of amniotic epithelial stem cells (AESCs) to keratinocytes (KCs). (**a**) Heatmap of 24 KCs-related TFs. (**b**) Intersection analysis among 24 KCs-related TFs and four epidermis-related GO terms enriched in KCs identified TP63 as the only overlapping factor. A: epidermis development; B: epidermis morphogenesis; C: regulation of epidermis development; D: skin epidermis development; E: KCs-related TFs. (**c**) TP63 overexpression in AESCs. (**d**) qRT-PCR analysis revealed significant increase of KCs-related stemness gene expressions in AESCs such as *KRT5*, *KRT14*, and *KRT15* after TP63 overexpression. (**e**) qRT-PCR analysis showing activation of Notch signaling and increase of differentiation genes in AESCs when cultured on Jagged 1-coated plates. FB, fibroblasts; AESC, amnion epithelial stem cell; KC, keratinocyte. Bars represent relative quantity normalized to GAPDH (mean ± SD) calculated from at least three independent experiments. Student’s *t-*test was used to calculate *p* values. Scale bar = 100 μm (**c**).

Considering the similarity between AESCs and KCs, cell fate convergence from AESCs to KCs would be a significant achievement. Cellular reprogramming through the overexpression of critical TFs allows the transdifferentiation of one type of somatic cell to another, providing further potential resources for regenerative medicine. Thus, 24 critical TFs for keratinocyte development were identified in the Cistrome Data Browser; transcriptome sequencing indicated that most of these showed low or negligible expression in AESCs (Figure 5a). A further intersection analysis among these 24 TFs and four epidermis-related GO terms enriched in KCs identified TP63 as the only overlapping factor (Figure 5b). As the master regulator of epidermal development and differentiation [29], TP63 is critical for maintaining the progenitor-cell populations [30]. To confirm the possibility of reprogramming AESC, TP63 was overexpressed in AESCs by lentiviral transduction (Figure 5c). It was found that the expression of KC undifferentiated markers such as *KRT5*, *KRT14*, and *KRT15* were significantly increased (Figure 5d). However, the levels of differentiation markers such as *KRT1* and *KRT10* were not increased.

In the human epidermis, Notch signaling is critical for initiating terminal differentiation of KCs [31]. To explore whether AESC differentiation features can be improved by activating Notch signaling, the Notch ligand Jagged 1 [32,33,34] was used. Jagged 1 elevated the expression of HES1, a direct target of the NOTCH pathway, and its downstream genes including *KRT1* and *INV* significantly (Figure 5e).

Overall, TP63 enhancement and NOTCH activation improved the undifferentiated features and differentiation capability of AESCs, respectively, and induced the cell fate convergence of AESCs to KCs.

## 4. Discussion

To promote wound healing in injured skin, various methods for KCs transplantation have been developed, including spray-based single cell delivery, cultured epithelial sheet, and tissue engineered skin. However, the translation to clinical practice is still challenging with KCs translation, and many issues, such as the cellular source, remain to be solved. Compared to adult cells, fetal stem cells offer more advantages for therapeutic applications in regeneration medicine. They show that fewer mutations accumulated over the lifetime, a greater self-renewal capability, multipotent differentiation potential [35], and better immunomodulatory properties [36]. Similarly, a previous study indicated that fetal KCs show faster expansion, longer telomeres, lower immunogenicity indicators, and greater clonogenicity compared to adult KCs [21]. However, clinical applications of fetal KCs are likely to be limited due to ethical considerations. After isolation from the placenta tissue, AESCs still maintain embryonic characteristics such as expressing stemness markers OCT4, NANOG, SSEA-4, etc [4] and differentiation capability [7]. Therefore, the present study not only revealed many commonalities between human AESCs and KCs using a transcriptome analysis and functional evaluation, but also revealed that, in stemness, AESCs show more similarities with early stage immature (fetal or neonatal) KCs compared to adult KCs. Thus, AESCs display several advantages in skin regeneration compared to allogeneic KCs, including (1) accessibility and high cell yield; (2) low immunogenicity; (3) noncontroversial source compared to fetal KCs; (4) more stemness and undifferentiated features compared to adult KCs. Since the bioengineering of fetal skin for clinical use remains an attractive prospect, our result may provide more evidence for the future application of AESCs to achieve this goal.

Epithelial cells are a specialized component in many organs. They are characterized by some common structural features, but have diverse functions that facilitate many specialized adaptations. The keratin family is a characteristic of distinct epithelial cells and show different expression patterns during cellular differentiation from embryonic to adult stages [19]. During embryonic development, keratins KRT8/KRT18 and KRT7/KRT19 appear earlier in single-layer multipotent epithelial cells [20,21,37] and are involved in placental barrier function [19]. Later, prior to stratification, these keratins are replaced with KRT5/KRT14 [38], marking the beginning of epidermal commitment [20]. KRT5/KRT14 are markers of the undifferentiated basal stem cell layer and parallel the proliferative capacity. They are usually absent from simple epithelia, with very few exceptions [39]. Both KRT1 and KRT10 are expressed when the epidermis starts to differentiate [40]. Our results revealed the intersections (both AESCs and KCs highly expressed KRT5 and KRT14) and the distinctions (AESCs highly expressed KRT7, KRT8, KRT18, and KRT19 while KCs highly express KRT1 and KRT10) between AESCs and adult KCs. Moreover, it is indicated that AESCs showed more similarity to immature KCs. All these results signify the dynamic process of epidermis development, stratification, and differentiation and also suggest the possibility of the same origin of AESCs and KCs.

In embryonic development, epithelial-to-mesenchymal transition (EMT) and mesenchymal-to-epithelial transition (MET) are evolutionarily conserved processes [41]. Cells can transition between epithelial and mesenchymal states in a highly plastic and dynamic manner [42]. In addition, the existence of an intermediate hybrid epithelial and mesenchymal phenotypes [43,44,45] confers a more plastic status on cancer cells. Mesenchymal properties of epithelial cells are often associated with their malignancy, including invasive behavior, cancer stem cell activity, and greater resistance to therapy [46]. Using transcriptome analysis and functional evaluation, the present study confirmed the co-expression of epithelial and mesenchymal features of AESCs, which is consistent with previous results [12,17]. The possession of mesenchymal features suggests more stemness with self-renewal and multipotency [43], which is essential for the maintenance of cell proliferative activity, undifferentiated status [47], and migratory capacity. In 2020, Richardson et al. proposed that AESCs that undergo EMT may move into the mesenchymal layer. They are retained as a pool of cells capable of repairing microfractures and gaps in the amnion membrane or recycling to the epithelium by undergoing MET [48]. Fibroblasts, the typical mesenchymal cells, show a strong potential in wound healing and in the injury repair of skin [49,50,51]. Therefore, the mesenchymal features of AESCs could also confer them with the capabilities to participate in these process.

The goal of regenerative medicine is to replace diseased tissues with functional cells or cellular products. Many studies have sought to generate KCs from fibroblasts [29,52], adipose tissue stem cells [53], and mesenchymal cells via reprogramming [54]. Due to its critical role in epidermal development [55], TP63 was once combined with other TFs and used for this purpose [29,54]. It has been shown that a combination of TP63 and KLF4 is sufficient to convert dermal fibroblasts to a keratinocyte phenotype in vitro [29]. Since KLF4 is already highly expressed in AESCs (data not shown), TP63 was used alone in this study and its overexpression was found to further increase levels of undifferentiated markers in AESCs. However, a high expression of TP63 counteracts the ability of Notch signaling in restricting growth and promoting differentiation [56], which may explain the lack of significant change in differentiation marker expression after reprogramming. As the differentiation capability of KCs is essential for the epidermal barrier function in human skin, the issue of whether AESCs are capable of differentiating is significant if applied for skin regeneration therapy in the future. It has been reported that activated Notch1 can induce the expression of differentiation markers including *KRT1*, *KRT10*, and *INV* [57]. Therefore, Jagged 1 was used in the present study to activate the Notch pathway, which assisted AESCs in improving the expression of these differentiation genes to some extent.

Although reprogramming with TP63 and Notch signaling activation mean that AESCs resemble KCs more closely, full convergence has not been achieved in this study, especially with respect to the differentiation capability. Thus, modified reprogramming strategies such as the use of small molecules may address this limitation and facilitate further modulation of the fate of human AESCs. In addition, transcriptome data of neonatal KCs published in a previous study were used here for comparison, which may have resulted in a bias. Future studies on fetal KCs may provide more information to identify their similarities with AESCs. Furthermore, single-cell RNA sequencing can also help to identify their characteristics more accurately and further enrich AESCs with specific features.

## 5. Conclusions

This study identified the similarity of AESCs to KCs, especially early stage immature KCs, and provided more supportive evidence for the use of AESCs in the construction of skin substitutes, especially fetal skin-like substitutes. Although differences exist, the undifferentiated and mesenchymal features which indicate more stemness and increased migratory capacity, could signify the superiority of AESCs in skin repair and regeneration; TP63 enhancement and NOTCH activation could facilitate the narrowing of the differences between AESCs and KCs. In future, modified reprogramming strategies, such as the use of small molecules, could facilitate a further modulation of the fate of human AESCs. The development of a feasible delivery system for AESCs is also required, and can help to address the issue of the cellular source for skin injury treatment in translational medicine.

## Figures and Tables

**Table 1:** Intersecting GO terms between AESCs and KCs.

Category	Description	AESCs-Up Gene Ratio	KCs-Up Gene Ratio
BP	Epidermis development	100/1644	160/2562
	Cell junction organization	62/1644	69/2562
	Morphogenesis of epithelium	73/1644	96/2562
	Regulation of epidermis development	20/1644	38/2562
	Molting cycle	23/1644	33/2562
	Hair cycle	23/1644	33/2562
	In utero embryonic development	46/1644	72/2562
CC	Cell–cell junction	93/1732	110/2666
	Cornified envelope	25/1732	42/2666
	Cell–cell adherens junction	30/1732	32/2666
	Cell cortex	52/1732	65/2666
MF	Cadherin binding	75/1659	112/2588
	Rho GTPase binding	38/1659	46/2588

BP, Biological Process; CC, Cellular Component; MF, Molecular Function.

## Data Availability

The RNA-seq data generated in this study have been deposited in the GEO database under the accession code: GSE182196. Publicly available genomic data sets used in this study include GSE147482 (human neonatal foreskin epidermis cells) and GSE127223 (DnKC, differentiated neonatal keratinocytes; nKC, neonatal keratinocytes).

## Supplementary Materials

The following supporting information can be downloaded at: https://www.mdpi.com/article/10.3390/cells11010070/s1, Figure S1: Heatmaps of overlapped genes highly expressed both in AESCs and KCs and involved in seven BP terms; Figure S2: Mean fluorescent intensity (MFI) of immunostaining; Figure S3: Skin reconstitution assay; Table S1: List of overlapped genes highly expressed both in AESCs and KCs and involved in seven BP terms.
