# Supplementary material for: Transcriptomic and Functional Evidence Show Similarities between Human Amniotic Epithelial Stem Cells and Keratinocytes
Source: Cells. 2021 Dec 27;11(1):70. doi: 10.3390/cells11010070 (PMC8750621; doi:10.3390/cells11010070)
Supplement: Supplementary file 1 [file cells-11-00070-s001.zip › cells-1478449-supplementary.pdf]

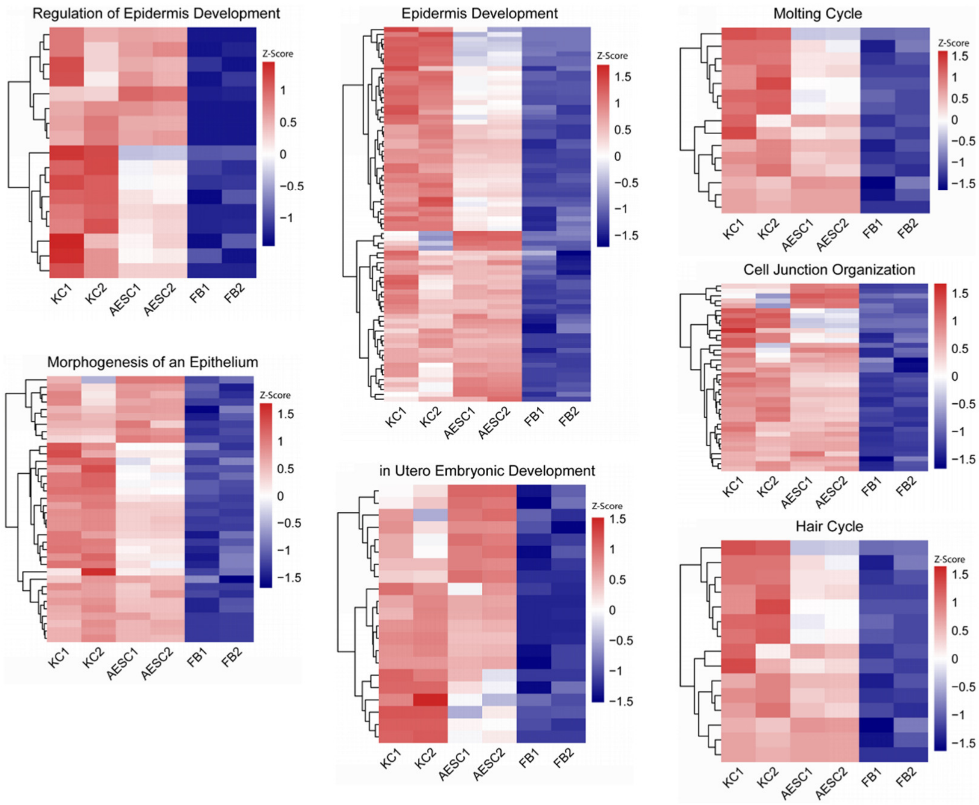

Figure S1. Heatmaps of overlapped genes highly expressed both in AESCs and KCs and involved in seven BP terms. KC, keratinocyte; AESC, amnion epithelial stem cell; FB, fibroblast.

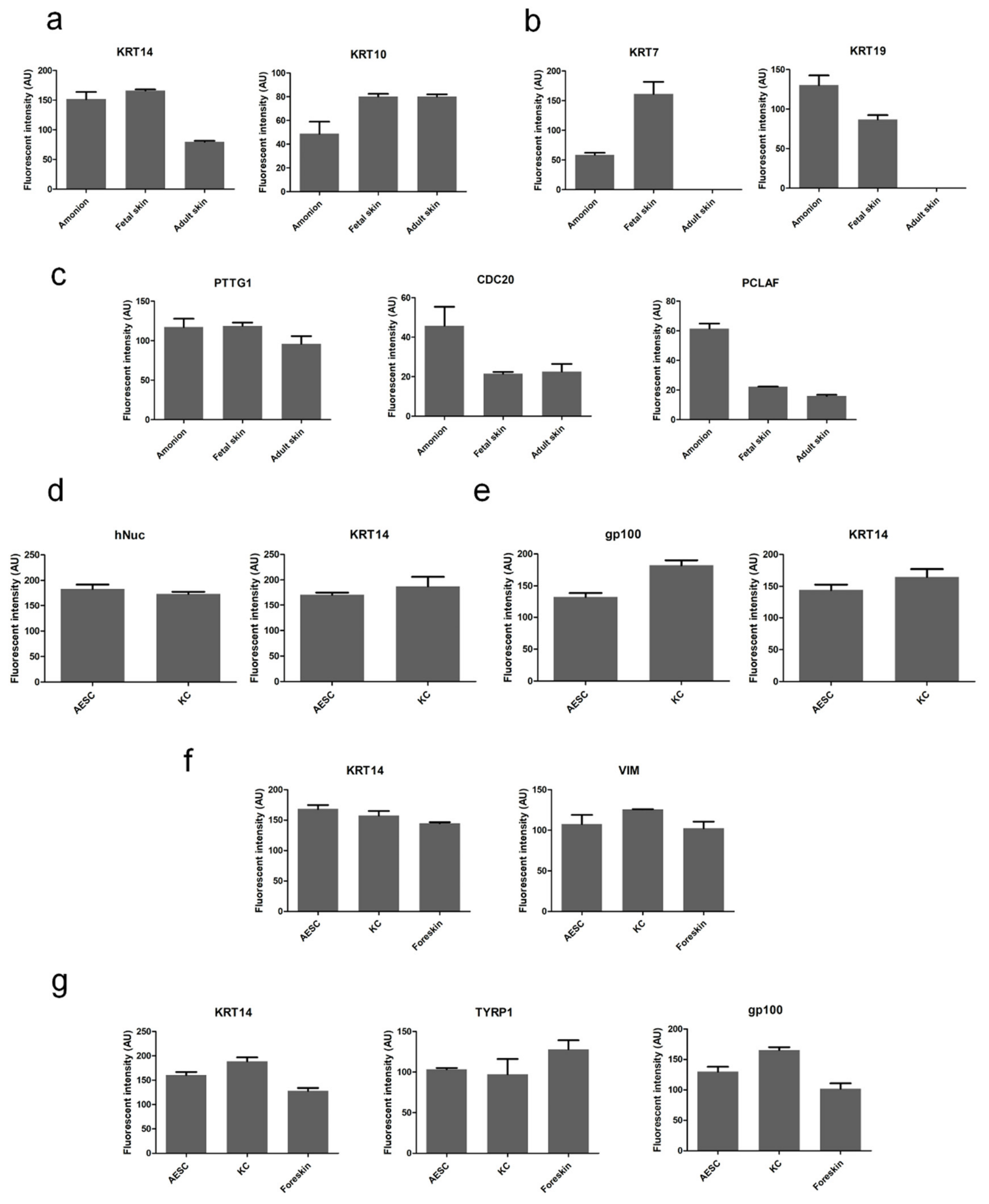

Figure S2. Mean fluorescent intensity (MFI) of immunostaining. (a) MFI of KRT14 and KRT10 in Figure 2c. (b) MFI of KRT7 and KRT19 in Figure 2d. (c) MFI of PTTG1, CDC20 and PCLAF in Figure 2f. (d) MFI of hNuc and KRT14 in Figure 3f. (e) MFI of gp100 and KRT14 in Figure 4b. (f) MFI of KRT14 and VIM in Figure 4d. (g) MFI of KRT14, TYRP1 and gp100 in Figure 4e and 4f.

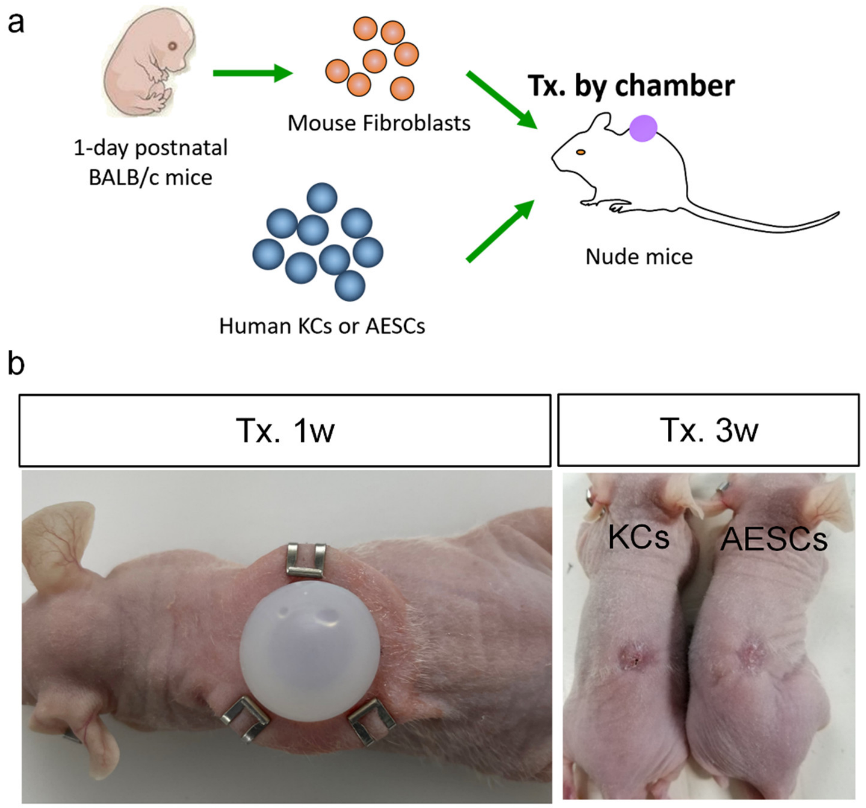

Figure S3. Skin reconstitution assay. (a) Flow chart of skin reconstitution assay using AESCs. (b) Photographs of BALB/c nude mice after transplantation with chamber at week 1 and week 3. KC, keratinocyte; AESC, amnion epithelial stem cell. Tx, transplantation.

Table S1. List of overlapped genes highly expressed both in AESCs and KCs and involved in seven BP terms

GO terms	Gene number	Gene name
Epidermis Development	77	*DSG2/KRT17/KRT14/JUP/PKP3/KRT5/COL17A1/LAMC2/LAMB3/FLNB/CELSR1/KRT16/ERRFI1/TGM1/FABP5/CDSN/DSG1/EVPL/SFN/SCEL/DSC3/DSG3/SPRR3/GRHL2/PRKCH/PKP1/DSC2/PERP/HPSE/ESRP1/LAMA5/PRSS8/ABCA12/KRT6C/GJB5/EPHA2/TMEM79/CST6/KRT8/SCRIB/KRT6A/SULT2B1/PKP2/KDF1/GRHL1/KRT18/TRIM16/IRF6/KLF4/POU3F1/OVOL2/KRT15/KRT6B/KRT13/SPRR1B/ST14/DSC1/ELF3/H2AFY/PCSK6/IVL/VANGL2/KLK7/CTSV/GAL/PTHLH/PPL/POU2F3/KRT23/SPRR1A/KRT78/KLK5/KRT9/FGFR2/SPRR2D/CDH3/OVOL1*
Morphogenesis of an Epithelium	36	*KRT17/SPINT2/MET/TACSTD2/CELSR1/SPINT1/EDN1/KRT16/TIAM1/GRHL2/GATA3/SERPINB5/LAMA5/EPHA2/TMEM79/CAMSAP3/SCRIB/RIPK4/ESRP2/DDR1/AREG/KDF1/COBL/TFAP2A/DVL1/WNT7A/KLF4/OVOL2/CELSR2/CTNNBIP1/SRC/ST14/IRX2/VANGL2/FGFR2/PAX8*
Regulation of Epidermis Development	17	*ERRFI1/CDSN/SFN/GRHL2/PRKCH/HPSE/ESRP1/TMEM79/SULT2B1/KDF1/GRHL1/TRIM16/OVOL2/H2AFY/CTSV/GAL/CDH3*
Cell Junction Organization	38	*DSG2/KRT14/JUP/PKP3/KRT5/COL17A1/LAMC2/LAMB3/ITGB4/ITGA6/LSR/F11R/RHOD/AFDN/DSG1/GRHL2/PRKCH/PKP1/PERP/NECTIN4/CDH1/RCC2/LAMA5/EPHA2/POF1B/CAMSAP3/PKP2/GRHL1/MARVELD3/MARVELD2/SRC/NECTIN1/PARD6B/F2RL1/INAVA/CRB3/CDH3/CXADR*
MoltingCycle	15	*KRT17/KRT14/CELSR1/KRT16/PER1/MPZL3/PPP1R13L/HPSE/LAMA5/TMEM79/TRPV3/VANGL2/GAL/FGFR2/CDH3*
Hair Cycle	15	*KRT17/KRT14/CELSR1/KRT16/PER1/MPZL3/PPP1R13L/HPSE/LAMA5/TMEM79/TRPV3/VANGL2/GAL/FGFR2/CDH3*
in Utero Embryonic Development	21	*SPINT2/SPINT1/MDFI/EDN1/ETNK2/DSC3/GRHL2/GJB3/GATA3/CHD7/GJB5/PDGFB/KRT8/OVOL2/ST14/ELF3/PLCD1/ASCL2/RRP7A/HOPX/FGFR2*
